# Inclusion of Cocoa Bean Shell in the Diet of Dairy Goats: Effects on Milk Production Performance and Milk Fatty Acid Profile

**DOI:** 10.3389/fvets.2022.848452

**Published:** 2022-02-18

**Authors:** Manuela Renna, Carola Lussiana, Letizia Colonna, Vanda Maria Malfatto, Antonio Mimosi, Paolo Cornale

**Affiliations:** ^1^Department of Veterinary Sciences, University of Torino, Grugliasco, Italy; ^2^Department of Agricultural, Forest and Food Sciences, University of Torino, Grugliasco, Italy

**Keywords:** agro-industrial by-products, caprine milk, lipids, production performance, *Theobroma cacao* (L.)

## Abstract

The use of agro-industrial by-products in animal nutrition is a promising strategy to reduce the food-feed competition, the diet cost at farm level and the environmental impact of animal-derived food production. In this study, the suitability of cocoa bean shell (CBS), a by-product of the cocoa industry, as a feed ingredient in the diet of dairy goats was evaluated, with a focus on the related implications on feed intake, milk yield, milk main constituents, and fatty acid (FA) profile of milk fat. Twenty-two Camosciata delle Alpi goats were divided into two balanced groups. All the goats were fed mixed hay *ad libitum*. The control group (CTRL; *n* = 11) also received 1.20 kg/head × day of a commercial concentrate, while in the experimental group (CBS; *n* = 11) 200 g of the CTRL concentrate were replaced by the same amount of pelleted CBS. The total dry matter intake of the goats was reduced by the dietary inclusion of CBS (*P* ≤ 0.01). The milk yield, as well as the milk fat, protein, and casein contents and yields were unaffected by the treatment. Milk from the CBS-fed goats showed decreased urea content when compared to the CTRL group (*P* ≤ 0.001). Milk from the CBS group of goats also showed increased concentrations of total branched-chain FA (both *iso* and *anteiso* forms; *P* ≤ 0.001) and total monounsaturated FA (*P* ≤ 0.05), as well as a decreased ∑ *n*6/∑ *n*3 FA ratio (*P* ≤ 0.05). *De novo* saturated FA, total polyunsaturated FA, total conjugated linoleic acids, and the majority of ruminal biohydrogenation intermediates remained unaffected by the dietary treatment. These results suggest that CBS can be strategically used as an alternative non-conventional raw material in diets intended for lactating goats, with no detrimental effects on their milk production performance. The use of CBS in goat nutrition may be hindered by the presence of theobromine, a toxic alkaloid. Special attention is needed by nutritionists to avoid exceeding the theobromine limits imposed by the current legislation. Detheobromination treatments are also suggested in literature to prevent toxic phenomena.

## Introduction

The world population is estimated to reach 9.7 billion in 2050 (1). Promoting a more efficient and sustainable use of the available resources has become a priority to satisfy the increasing global food demand (2). Agro-industrial by-products are addressed as innovative raw materials to be used in feed formulations for livestock animals to reduce both the human-animal competition for food supply and the animal feeding costs (3).

Cocoa beans (CB) are the seeds of the cocoa tree (*Theobroma cacao* L., botanical family: Sterculiaceae). They are composed of an outer shell, two cotyledons and a small germ (4). In 2018/2019, the total worldwide CB production was approximately equal to 4.8 million tons (5), with a forecast of 5% growth for the 2020/2021 period. Africa is the leader cocoa producer worldwide, covering the 76% of total production. Significant contributions to the world cocoa trade are also given by Americas, Indonesia, and Papua New Guinea (18.0, 4.6, and 0.6% of the total production, respectively) (5).

Cocoa beans are destined to the confectionery industry to produce chocolate and other cocoa-derivative foods (e.g., cocoa paste, butter, and liquor) that are largely consumed in the most developed countries, such as Europe and North America (6). Only the 10% of the total weight of the fruit is commercialized and, consequently, different by-products are originated (7). After the harvesting and post-harvesting processes, the removal of CB causes the discarding of the cocoa pod husk, a first by-product representing the 70–75% of the cocoa fruit weight, and of the cocoa mucilage, a white mass surrounding the CB (6, 8). During the winnowing and roasting processes, the cocoa bean shell (CBS), which represents about the 10–17% of the total bean weight (9), is removed from the seeds (4, 7). It can be estimated that about 900,000 tons of CBS waste are produced annually worldwide (10). On a dry matter (DM) basis, the CBS contains 10.3–27.4% of protein, 39.3–66.3% of fiber, and 1.5–8.5% of fat (7). The CBS is also a natural source of bioactive compounds, like polyphenols (total phenolic content ranging from 3.1 to 95.0 mg of gallic acid equivalents/g of dried CBS) (7), with antioxidant and antiradical properties (4, 11).

To reduce waste and maximize the principles of Circular Economy (12), recent studies have been carried out to evaluate the potential uses of CBS. For food purposes, CBS has been mainly proposed as a low-cost cocoa substitute (7), that is as ingredient or additive in baked products (13–15). CBS has also been successfully used to produce alkalized fiber powder (16). In addition, polyphenols extracted from CBS were shown to improve the quality parameters of cooking oils (17) and to reduce lipid oxidation of cooked-refrigerated beef (18). Besides food applications, CBS can also be employed for biofuel production, thanks to its high calorific value (19). Studies were published showing the possibility to produce biomethane (20) and biogas from CBS (21). Thanks to its physical properties (22), antioxidant and biodegradable characteristics, CBS has been also used in food packaging (23, 24), cosmetics and biomedical applications (25), farm bedding material (26), and as fertilizer (7).

In recent years, CBS has also been targeting the livestock sector as a feedstuff (27). The main limitation in the use of CBS as feed ingredient is the presence of theobromine (3,7-dimethylxanthine), an alkaloid prone to cause toxicity and feed intake reduction at high dietary concentrations (28), the levels of which can be decreased by means of hot water, alkali, microbial, or enzymatic detheobromination treatments (28–30). The European Food Safety Authority established a maximum level of theobromine in animal feed equal to 300 mg/kg (31). Currently, exceptions are provided for pigs (200 mg/kg), and for dogs, rabbits, horses, and fur animals (50 mg/kg) (32). Ogunsipe et al. (33) replaced up to 40% maize with CBS in the diet of weaner pigs, showing no deleterious effects on live weight gain and feed conversion at inclusion levels lower than 20 and 10%, respectively. Adeyina et al. (30) and Ayinde et al. (34) demonstrated that untreated CBS and hot water treated CBS can be included in the diet of weaner rabbits at 100 and 200 g/kg, respectively, with no negative effects on animal performance and physiological responses, with the additional benefit of increased farm profitability (34). Studies on poultry nutrition showed that hot water treated CBS and enzyme treated CBS can replace maize up to 20 and 10% in the diet of commercial layers, respectively, without any detrimental effects on the performance, hematology, and egg quality parameters (29, 35). In the aquaculture sector, Bamba et al. (36) demonstrated that CBS, when used in combination with a mixture of plant proteins, is a suitable feed for Nile tilapia, being able to improve the weight gain and the feed conversion ratio of the fish. As far as ruminants are concerned, Amin and Cahyono (37) showed increased daily body weight gain and feed efficiency in young male Bali cattle fed king grass and a protein supplement consisting of 40% cocoa bean waste (a mixture of bean skin, pulp, and placenta) plus 60% rice bran compared to a supplement consisting of 100% rice bran. When included at about 12% in diets for lactating ewes to partially replace corn and barley, CBS did not modify milk yield, milk fat, and protein contents, while it reduced the milk urea content and improved the health indexes of milk fat (38).

No information is currently available on the suitability of CBS as feed ingredient in the diet of dairy goats. Therefore, this study aimed at providing novel information on the effects of the dietary inclusion of CBS on feed intake, milk yield, milk main constituents, and fatty acid (FA) profile of milk fat from lactating goats.

## Materials and Methods

The experimental protocol was designed according to the guidelines of the current European Directive 2010/63/EU on the protection of animals used for scientific purposes. All procedures and treatments were in compliance with the European Directive (Council Directive 98/58/EC) on the minimum standards for the protection of animals bred or kept for farming purposes.

### Animals and Dietary Treatments

The experiment was carried out in a commercial farm located in None (Turin province, Piedmont, NW Italy, latitude: 44°91′59″ N; longitude: 07°52′57″ E; altitude: 270 m a.s.l.) from May 14 to June 13, 2019. Twenty-two pluriparous Camosciata delle Alpi goats in mid-lactation were selected from a flock of 60 lactating goats and allocated to two balanced groups of 11 animals each, according to their parity (3.9 ± 1.07), stage of lactation (106 ± 4.6 days in milk), milk yield (1.8 ± 0.06 kg/head × day), milk gross composition (fat: 33.8 ± 0.43 g/kg and protein: 32.3 ± 0.42 g/kg) and FA profile of milk fat. The groups were then randomly assigned to a control or an experimental diet, with 55:45 forage to concentrate (F:C) ratio on a DM basis. The experimental diets were formulated following NRC recommendations (39). The control group (CTRL) was fed mixed hay *ad libitum* and 1.20 kg/head × day of a pelleted commercial concentrate containing corn (34.0%), partially dehulled sunflower meal (15.0%), dehulled soybean meal (12.0%), wheat bran (11.0%), barley (10.0%), soybean hulls (4.0%), roasted soybean (4.0%), dehydrated alfalfa (3.0%), cane molasses (2.5%), calcium carbonate (2.0%), sodium chloride (1.0%), precipitated dicalcium phosphate dihydrate (0.5%), sodium bicarbonate (0.3%), magnesium oxide (0.2%), and mineral-vitamin premix (0.5%). The concentrate was administered in equal amounts during the milking, at 6:30 a.m. and 6:00 p.m. The other group (CBS group, Cocoa Bean Shell) received the same diet but, during the afternoon milking, 200 g of concentrate were replaced by pelleted CBS (which approximately corresponded to the 17% of the daily administered concentrate). The dietary CBS inclusion level was chosen such as to fall within the maximum threshold imposed by the current European regulation on the presence of theobromine in animal feeds (32), in the meantime allowing to formulate isonitrogenous and isoenergetic experimental diets. The goats received increasing quantity of CBS (diet adaptation) starting 10 days before the beginning of the trial: 50 g/head in the 1st day, 100 g in the next 2 days, then 150 g in other 2 days and 200 g/head × day from the 6th day to the end of the trial.

All the selected goats were housed indoors in individual pens and had free access to clean and fresh water.

### Feed Intake, Sampling, and Analysis

Feed refusals were weighed daily to estimate feed intake. Representative samples of hay, concentrate and CBS used in the trial were collected at the beginning of the adaptation period. They were ground with a cutting mill to pass a 1 mm screen sieve (Pulverisette 15-Fritsch GmbH, Idar-Oberstein, Germany). AOAC International procedures (40) were used to determine DM (method no. 930.15), ash (method no. 942.05), crude protein (CP, method no. 984.13), acid detergent fiber and acid detergent lignin (ADFom and ADL, method no. 973.18). Ether extract (EE, method no. 2003.05) was analyzed according to AOAC International (41). Neutral detergent fiber (aNDFom) was analyzed according to Mertens (42); α-amylase (Merck, Darmstadt, Germany) and sodium sulphite (Merck, Darmstadt, Germany) were added, and results were corrected for residual ash content.

Rumen degradable protein (RDP) was analyzed according to Licitra et al. (43).

The energetic value of feeds was expressed as net energy for lactation (NE_L_) and was estimated using National Research Council equations (39).

The FA composition of hay, concentrate and CBS was assessed using a combined direct transesterification and solid-phase extraction method as reported in Dabbou et al. (44). Separation, identification, and quantification of fatty acid methyl esters (FAME) were performed as described by Renna et al. (45). The results were expressed as g/kg DM. The daily intake (g/head) of each individual FA and groups of FA from the diet was estimated considering the daily intake and the analytically determined FA composition of each feedstuff.

The contents of total extractable phenols (TEP) and different polyphenol fractions (non-tannin phenols, NTP; condensed tannins, CT) in CBS, were assessed as detailed in Iussig et al. (46). Briefly, polyphenols were extracted twice with aqueous acetone (70:30 v/v) and subjected to ultrasonic treatment for 20 min at room temperature in an ultrasonic water bath (Bransonic-21, Branson Ultrasonics, Danbury, CT, USA). Polyvinyl-polypyrrolidone was used to separate NTP and total tannins (TT), according to a modified Folin-Ciocalteu method. The butanol-HCl-iron method was used to determine CT. The absorbance was recorded at 725 nm (TEP and NTP, expressed as gallic acid equivalents) and 550 nm (CT, expressed as leucocyanidin equivalents) using a UV–vis spectrophotometer (Shimadzu UVmini-1240, Shimadzu Corporation, Kyoto, Japan). Total tannins (TT) were computed as the difference between TEP and NTP. Hydrolysable tannins (HT) were estimated as the difference between TT and CT (47). The amounts of phenolic compounds daily ingested by the goats belonging to the CBS group was estimated based on the analyzed phenolic composition of CBS and the determined intake of concentrate.

All the analyses were performed in duplicate.

### Milk Sampling and Analysis

The goats were milked twice a day (at 6:30 a.m. and 6:00 p.m.) in a 12-unit parallel milking parlor (Milkline®, Piacenza, Italy) with 43 kPa vacuum level combined with 90 cycles/min of pulsation rate. Individual milk yield was recorded and individual composite samples (*n* = 88) of morning and afternoon milking (proportional to milk production recorded per milking) were collected at 0, 10, 20, and 30 days after the beginning of the trial. Each milk sample was divided into two aliquots, immediately stored at 4°C in a portable refrigerator and transported to the laboratory. The first aliquot (50 mL) was analyzed for fat, protein, lactose, casein, and urea (MilkoScan FT 6000, Foss Electric, Hillerød, Denmark; ISO 9622:2013/IDF 141:2013) and somatic cell count (SCC) (Fossomatic FC, Foss Electric, Hillerød, Denmark; UNI EN ISO 13366-2:2007). The second aliquot (150 mL) was frozen at −80°C and successively analyzed for the FA composition. Milk fat extraction was obtained by centrifugation at 7,300 rpm for 30 min at −4°C. The resulting molten butter was filtered through a hydrophobic filter (Whatman 1, Whatman International Ltd., Maidstone, England). The pure milk fat was then dissolved in 5 mL of internal standard solution (nonanoic acid in heptane) and FAME were obtained by transesterification of glycerides by using a solution of potassium hydroxide in methanol, as reported in Cornale et al. (48). FAME were analyzed by high-resolution gas chromatography (Shimadzu GC 2010 Plus, Shimadzu Corporation Analytical Instruments Division, Kyoto, Japan) equipped with a flame ionization detector. As reported in Kramer et al. (49), FAME were separated on a CP-Sil 88 fused capillary column (100 m × 0.25 mm ID, 0.20 μm film thickness; Varian Inc., Lake Forest, CA, USA). The temperatures of both injector and detector were maintained at 250°C. The temperature program was: 45°C for 4 min, raised to 175°C at 13°C/min for 27 min, then to 215°C at 4°C/min for 35 min. Carrier gas was used at a flow rate of 1 ml/min. The injection volume was 0.1 μL. Peaks were identified by comparing their retention times with pure FAME standards (Matreya Inc., Pleasant Gap, PA, USA and Restek Corporation, Bellefonte, PA, USA) and by comparison with published chromatograms (49).

Energy-corrected milk (ECM) was calculated for each goat using the following formula (50):


$$\begin{array}{l} ECM\;\left(kg\right)=\left[\left(kg\;of\;milk\right)\times 0.327\right]+\left[\left(kg\;of\;fat\right)\times 12.925\right]\\ +\left[\left(kg\;of\;protein\right)\times 7.2\right]. \end{array}$$

Feed efficiency (FE) is defined as a unit of milk produced per unit of DM consumed and was calculated as reported in Renna et al. (51) as the ratio between ECM (kg) and dry matter intake (DMI, kg).

The activity of the enzyme Stearoyl-CoA desaturase (SCD; EC 1.14.19.1) was estimated by the calculation of different desaturase indexes: DI_14_ (C14:1 *c*9/C14:0), DI_16_ (C16:1 *c*9/C16:0) and DI_18_ (C18:1 *c*9/C18:0).

All the analyses were performed in duplicate.

### Statistical Analysis

The changes in DMI, milk yield, milk composition, and FE were analyzed using the MIXED procedure of SAS v. 9.4 (SAS Inst. Inc, Cary, NC) for repeated measures over time. The goat was considered as the experimental unit. Compound symmetry, first order autoregressive or unstructured covariance structure, according to the smallest Schwarz Bayesian information criterion, was applied (52). The following model was used:


$$\begin{array}{l} Y_{ijk}=\;\mu +DT_{i}+G_{\left(i\right)j}+ST_{k}+{\left(DT\times ST\right)}_{jk}+\varepsilon_{ijk} \end{array}$$

where: *Y*_*ijk*_= mean of response variable, μ= population mean, *DT*_*i*_ = fixed effect of dietary treatment, *G*_(*i*)*j*_ = random effect of goat within the treatments, *ST*_*k*_= fixed effect of sampling time, (*DT* × *ST*)_*jk*_= fixed effect of interaction between dietary treatment and sampling time, and ε_*ijk*_= experimental error. Significance was declared at *P* ≤ 0.05. The results of the statistical analysis are reported as estimate least-squares means.

## Results

### Chemical Composition of Cocoa Bean Shell and Experimental Diets

The proximate and FA compositions of the feedstuffs and of the experimental diets are shown in Table 1. The CBS used in this trial showed high CP (173 g/kg DM) and fiber (aNDFom: 495 g/kg DM; ADFom: 426.4 g/kg DM) concentrations, while the EE concentration was relatively low (60.6 g/kg DM). The NE_L_ of the by-product was equal to 4.27 MJ/kg DM. The experimental diets were almost isonitrogenous (143 and 141 g/kg DM for the CTRL and CBS diets, respectively) and isoenergetic (5.31 and 5.15 MJ/kg DM for the CTRL and CBS diets, respectively).

**Table 1:** Proximate composition (g/kg DM, unless otherwise stated) and fatty acid profile (g/kg DM) of the experimental feedstuffs.

**Parameter**	**Experimental feedstuffs**			**Dietary treatment**	
	**Hay**	**Concentrate**	**Cocoa bean shell**	**CTRL**	**CBS**
**Main nutrients (g/kg DM, unless otherwise stated)**					
DM (g/kg)	890	884	898	887	888
Ash	73	119	92	93	91
CP	93	206	173	143	141
RDP (% CP)	60	49	41	55	54
EE	25	34	61	28	31
aNDFom	684	231	495	483	502
ADFom	411	124	426	283	306
ADL	58	22	177	42	54
NSC^a^	126	409	179	251	234
NE_L_ (MJ/kg DM)	4.34	6.54	4.27	5.31	5.15
**Fatty acids (g/kg DM)**					
C12:0	0.07	0.04	0.10	0.05	0.06
C14:0	0.09	0.10	0.38	0.09	0.12
C16:0	1.79	6.78	13.46	4.46	5.31
C16:1 *t*3	0.11	0.03	0.05	0.07	0.06
C16:1 *c*9	0.02	0.06	0.50	0.04	0.08
C18:0	0.15	1.68	13.71	0.97	2.06
C18:1 *c*9	0.23	9.17	15.16	5.02	6.03
C18:1 *c*11	0.03	0.37	0.87	0.21	0.28
C18:2 *c*9*c*12	0.88	13.72	4.20	7.76	7.71
C20:0	0.07	0.16	0.58	0.12	0.16
C18:3 *c*6*c*9*c*12	0.01	0.01	0.02	0.01	0.01
C20:1 *c*9	0.02	0.11	0.05	0.07	0.07
C18:3 *c*9*c*12*c*15	1.27	1.11	0.34	1.19	1.11
C22:0	0.17	0.13	0.30	0.15	0.16
C20:3 n6	n.d.	n.d.	0.17	0.00	0.01
C20:4 n6	n.d.	n.d.	0.10	0.00	0.01
C20:5 n3	n.d.	n.d.	0.25	0.00	0.02
Σ SFA	2.33	8.89	28.54	5.85	7.86
Σ MUFA	0.41	9.74	16.62	5.41	6.52
Σ PUFA	2.17	14.84	5.08	8.96	8.87
TFA	4.91	33.47	50.23	20.22	23.25

*DM, dry matter; CP, crude protein; RDP, rumen degradable protein; EE, ether extract; aNDFom, neutral detergent fiber; ADFom, acid detergent fiber; ADL, acid detergent lignin; NSC, non-structural carbohydrates; NE_L_, net energy for lactation; n.d., not detected; c, cis; t, trans; SFA, saturated fatty acids; MUFA, monounsaturated fatty acids; PUFA, polyunsaturated fatty acids; TFA, total fatty acids*.

a *Calculated as: 1,000 − (aNDFom + CP + EE + ash)*.

As far as the FA composition is concerned, CBS showed a prevalence of saturated fatty acids [SFA; 28.54 g/kg DM, corresponding to the 56.8% of total detected FA (TFA)] and of monounsaturated fatty acids (MUFA; 16.62 g/kg DM, corresponding to the 33.1% of TFA). Palmitic (C16:0) and stearic (C18:0) acids were the most abundant SFA in CBS, showing approximately the same concentration (13.46 and 13.71 g/kg DM, respectively) in the by-product (representing the 47.2 and 48.0% of total SFA and 26.8 and 27.3% of TFA, respectively). Oleic acid (C18:1 *c*9) was the most abundant individual FA in CBS (representing the 91.2% of total MUFA and 30.2% of TFA). Polyunsaturated fatty acids (PUFA) only represented a minor component of CBS (5.08 g/kg DM), comprising the 10.1% of TFA. Linoleic acid (C18:2 *c*9*c*12) was the most abundant PUFA in the by-product, accounting for the 8.4% of TFA (82.7% of total PUFA). Alpha-linolenic acid (C18:3 *c*9*c*12*c*15) was only detected in very low amounts in CBS (6.7% of PUFA and 0.7% of TFA). The TFA content of the experimental diets was higher for the CBS when compared to the CTRL diet (23.25 and 20.22 g/kg DM, respectively). However, the diets were very similar in terms of their FA composition, with the CBS diet showing a slightly higher concentration of total SFA (7.86 vs. 5.85 g/kg DM, corresponding to 33.8 and 28.9% of TFA) and total MUFA (6.52 vs. 5.41 g/kg DM, corresponding to 28.0 and 26.8% of TFA) and a very similar concentration of total PUFA (8.87 vs. 8.96 g/kg DM, corresponding to 38.2 and 44.3% of TFA) when compared to the CTRL diet.

Regarding phenols (data not reported in the table), the recorded amount of TEP in CBS was low (9.12 g/kg DM). More than the 96% of TEP was represented by tannins (TT equal to 8.76 g/kg DM), almost equally represented by hydrolysable (HT) and condensed (CT) forms (55.1 and 44.9% of TT, respectively). Polyphenols were not determined in mixed hay and CTRL concentrate, as their amounts were expected to be negligible.

### Intake of Dry Matter, Fatty Acids, and Phenolic Compounds

The effects of the dietary inclusion of CBS on DM and FA intake by the goats are shown in Table 2. The dietary treatment had no significant effect on the individual intake of concentrate, which was always totally ingested by the goats in both experimental groups. However, a significant decrease of the individual intake of hay was observed with the inclusion of CBS in the diet (1.22 kg/head × day and 1.11 kg/head × day for the CTRL and CBS diets, respectively; *P* ≤ 0.001). Consequently, the individual total DMI of the goats was significantly reduced with the dietary inclusion of CBS (−5%; *P* ≤ 0.01).

**Table 2:** Dry matter and main fatty acids intakes of goats fed the control (CTRL) and cocoa bean shell (CBS) diets^a^.

**Parameter**	**Dietary treatment**		**Effects^b^**	
	**CTRL**	**CBS**	**DT**	**SD**
**DM intake (kg/head** **×day)**				
Hay	1.22	1.11	***	***
Concentrate	1.06	1.06	–^c^	–^c^
Total	2.28	2.16	**	***
**FA intake (g/head** **×day)**				
C12:0	0.12	0.13	*	***
C14:0	0.21	0.25	***	***
C16:0	9.34	10.37	***	***
C16:1 *t*3	0.17	0.16	***	***
C16:1 *c*9	0.09	0.16	***	***
C18:0	1.95	4.11	***	***
C18:1 *c*9	9.96	11.05	***	***
C18:1 *c*11	0.43	0.52	***	***
C18:2 *c*9*c*12	15.56	13.81	***	***
C20:0	0.26	0.33	***	***
C18:3 *c*6*c*9*c*12	0.23	0.24	*	***
C20:1 *c*9	0.14	0.12	***	***
C18:3 *c*9*c*12*c*15	2.73	2.45	***	***
C22:0	0.34	0.36	**	***
C20:3 n6	n.d.	0.03	–	–
C20:4 n6	n.d.	0.02	–	–
C20:5 n3	n.d.	0.05	–	–
ΣSFA	12.23	15.54	***	***
Σ MUFA	10.79	12.02	***	***
Σ PUFA	18.31	16.37	***	***
TFA	41.33	43.93	***	***

*CTRL, control diet; CBS, cocoa bean shell diet; DT, dietary treatment; SD, sampling date; DM, dry matter; FA, fatty acids; c, cis; t, trans; SFA, saturated fatty acids; MUFA, monounsaturated fatty acids; PUFA, polyunsaturated fatty acids; TFA, total fatty acids*.

a *Total number of measurements collected for each group equal to 341 (11 goats × 31 sampling days)*.

b Probability: ***P ≤ 0.001; **P ≤ 0.01; *P ≤ 0.05; ns, not significant (P > 0.05). The effect of interaction between dietary treatment and sampling date (DT × SD) was not significant; therefore, significance is only presented for main effects.

c *During the measurement period, all the offered concentrate was eaten by the goats with no refusals*.

Table 2 also shows the differences between the two groups of goats for the intake of all individual FA and groups of FA from the diets. In particular, the CBS group of goats showed a higher intake of palmitic acid (+ 1.03 g/head × day; *P* ≤ 0.001), stearic acid (+ 2.16 g/head × day; *P* ≤ 0.001) and oleic acid (+ 1.09 g/head × day; *P* ≤ 0.001), as well as contemporarily lower intakes of linoleic acid (−1.75 g/head × day; *P* ≤ 0.001) and α-linolenic acid (−0.28 g/head × day; *P* ≤ 0.001) when compared to the CTRL group. Overall, the CBS-fed goats ingested higher amounts of total SFA and total MUFA, and lower amounts of total PUFA, that the CTRL-fed goats (in all cases, *P* ≤ 0.001).

The amounts of TEP, NTP, TT, CT, and HT daily ingested by the goats belonging to the CBS group were equal to 1.64, 0.06, 1.57, 0.61, and 0.87 g/head × day, respectively.

The DT × SD interaction was not significant for the considered parameters.

### Milk Yield and Milk Main Constituents

The effects of the dietary inclusion of CBS on milk yield, ECM, milk fat, protein, casein and lactose contents and yields, urea content, SCC, and FE are shown in Table 3. The individual milk yield and ECM were not significantly affected by the treatment. Regarding the main milk constituents, both the content (−3.9%; *P* < 0.001) and yield (−8.8%; 0.05 < *P* ≤ 0.10) of lactose as well as the urea content (−20.7%; *P* < 0.001) were reduced or tended to be reduced with the inclusion of CBS in the diet. The other macro nutrients in milk (fat, protein, and casein), as well as SCC and FE showed no differences while comparing the experimental groups.

**Table 3:** Milk yield, milk main constituents, and somatic cell count of milk from goats fed the control (CTRL) and cocoa bean shell (CBS) diets^a^.

**Parameter**	**Dietary treatment**		**Effects^b^**	
	**CTRL**	**CBS**	**DT**	**SD**
Milk yield (kg/head × day)	1.81	1.70	ns	ns
ECM^c^ (kg/head × day)	1.84	1.75	ns	ns
**Milk composition (g/kg)**				
Fat	37.37	37.05	ns	ns
Protein	32.04	33.44	ns	ns
Casein	24.76	26.01	ns	ns
Lactose	41.39	39.76	***	*
**Component yield (g/head** **× day)**				
Fat	65.59	61.25	ns	ns
Protein	56.10	55.29	ns	ns
Casein	43.20	43.03	ns	ns
Lactose	74.67	68.08	†	ns
Urea (mg/dL)	48.01	38.09	***	***
SCC (×1,000 cells/mL)	942.49	1,096.37	ns	ns
FE^d^	0.81	0.81	ns	ns

*CTRL, control diet; CBS, cocoa bean shell diet; DT, dietary treatment; SD, sampling date; ECM, energy corrected milk; SCC, somatic cell count; FE, feed efficiency*.

a *Total number of samples analyzed for each group equal to 44 (11 goats × 4 sampling days)*.

b Probability: ***P ≤ 0.001; *P ≤ 0.05; ^†^0.05 < P ≤ 0.10; ns, not significant (P > 0.05). The effect of the interaction between dietary treatment and sampling date (DT × SD) was not significant; therefore, significance is only presented for the main effects.

c *ECM = [0.327 × milk (kg/day)] + [12.95 × milk fat (kg/day)] + [7.2 milk protein (kg/day)] (50)*.

d *Calculated as: ECM (kg)/dry matter intake (kg) (50)*.

The DT × SD interaction was not significant for the considered parameters.

### Milk Fatty Acid Profile

The inclusion of CBS in the diet significantly affected some individual FA and groups of FA in goat milk fat (Tables 4–**6**). The concentration of the majority of individual SFA with a linear carbon chain, as well as total SFA, did not significantly vary between the experimental groups, with few exceptions (Table 4). The latter regarded heptanoic acid (C7:0), which was negatively affected by the inclusion of CBS in the goat diet (*P* ≤ 0.01), and some long chain SFA, such as C18:0, eicosanoic acid (C20:0) and behenic acid (C22:0), which showed significantly higher concentrations in the milk from CBS when compared to the milk from the CTRL group (*P* ≤ 0.05, *P* ≤ 0.001, and *P* ≤ 0.001, respectively). Considering SFA, most changes were instead detected in the milk concentrations of branched-chain fatty acids (BCFA), both *iso* and *anteiso* form (Table 4). Total BCFA, total *iso* BCFA and total *anteiso* BCFA were, in fact, significantly enhanced (by 10.9, 10.0, and 13.8%, respectively) in the milk from CBS goats when compared to the CTRL group (*P* ≤ 0.001). Considering individual BCFA, significant increases in CBS milk were observed for C14 *iso*, C15 *iso*, C16 *iso*, C15 *anteiso* and C17 *anteiso*; C13 *iso* showed a tendency toward higher concentrations in CBS than CTRL milk, while C13 *anteiso*, C17 *iso*, and C18 *iso* remained unaffected by the dietary treatment.

**Table 4:** Saturated and branched-chain fatty acids (g/kg fat) in milk of goats fed the control (CTRL) and cocoa bean shell (CBS) diets^a^.

**Fatty acid (g/kg fat)**	**Dietary treatment**		**Effects^b^**	
	**CTRL**	**CBS**	**DT**	**SD**
C4:0	1.49	1.57	ns	ns
C6:0	2.10	2.09	ns	***
C7:0	0.03	0.02	**	**
C8:0	2.30	2.29	ns	***
C10:0	7.44	7.26	ns	***
C11:0^c^	0.26	0.24	ns	***
C12:0	3.19	3.26	ns	***
C13:0	0.07	0.07	ns	***
C14:0	8.00	8.14	ns	***
C15:0	0.85	0.85	ns	***
C16:0	21.50	20.25	†	***
C17:0	0.54	0.56	ns	***
C18:0	8.04	8.83	*	***
C19:0^d^	0.07	0.07	ns	*
C20:0	0.16	0.18	***	***
C22:0	0.06	0.07	***	**
C13:0 *iso*	0.031	0.033	†	***
C13:0 *anteiso*	0.04	0.04	ns	***
C14:0 *iso*	0.10	0.13	***	***
C15:0 *iso*	0.24	0.27	**	***
C15:0 *anteiso*	0.34	0.40	***	***
C16:0 *iso*	0.24	0.27	**	***
C17:0 *iso*	0.35	0.36	ns	***
C17:0 *anteiso*	0.34	0.38	**	***
C18:0 *iso*	0.04	0.04	ns	ns
Σ SFA	57.75	57.37	ns	***
Σ BCFA	1.73	1.92	***	***
Σ *iso* BCFA	1.00	1.10	***	***
Σ *anteiso* BCFA	0.72	0.82	***	***

*CTRL, control diet; CBS, cocoa bean shell diet; DT, dietary treatment; SD, sampling date; SFA, saturated fatty acids; BCFA, branched chain fatty acids*.

a *Total number of samples for each group equal to 44 (11 goats × 4 sampling dates)*.

b Probability: ***P ≤ 0.001; **P ≤ 0.01; *P ≤ 0.05; ^†^ 0.05 < P ≤ 0.10; ns, not significant (P > 0.05). The effect of the interaction between dietary treatment and sampling date (DT × SD) was not significant; therefore, significance is only presented for the main effects.

c *Coeluted with C10:1 c9*.

d *Coeluted with C18:1 c15*.

Differently from what observed for the total SFA, the concentration of the total MUFA significantly increased (+5.1%; *P* < 0.05) in the milk from CBS-fed goats when compared to the milk from CTRL-fed goats (Table 5). The concentration of total octadecenoic acids tended to increase significantly with the inclusion of CBS in the goat diet (*P* = 0.08) while the total trans-octadecenoic acids remained unaffected. Significant increases were observed for the concentrations of oleic acid (that with the applied chromatographic conditions coeluted with C18:1 *c*10 and C18:1 *t*15, + 5.1%; *P* ≤ 0.05) and other individual octadecenoic isomers (i.e., C18:1 *c*11 and C18:1 *c*14, + 7.1% *P* ≤ 0.05 and + 16.7% *P* ≤ 0.01, respectively). Significant increases were also recorded for the concentrations of C16:1 *c*13, C17:1 *t*9 and C20:1 *t*6-11, while C16:1 *t*4 decreased with the inclusion of CBS in the diet. The other individual detected MUFA were not affected by the treatment. The SCD activity, as estimated by DI_14_, DI_16_, and DI_18_, did not vary between the treatments.

**Table 5:** Monounsaturated fatty acids (g/kg fat) in milk of goats fed the control (CTRL) and cocoa bean shell (CBS) diets^a^.

	**Dietary treatment**		**Effects^b^**	
**Fatty acid (g/kg fat)**	**CTRL**	**CBS**	**DT**	**SD**
C12:1 *c*9	0.08	0.08	ns	***
C14:1 *c*9	0.12	0.12	ns	***
C14:1 *t*9	0.01	0.01	ns	*
C16:1 *c*7	0.24	0.24	ns	**
C16:1 *c*9	0.43	0.41	ns	***
C16:1 *c*10	0.01	0.01	ns	***
C16:1 *c*11	0.01	0.01	ns	*
C16:1 *c*12	0.14	0.13	ns	***
C16:1 *c*13	0.005	0.007	***	ns
C16:1 *t*4	0.020	0.015	**	***
C16:1 *t*5	0.01	0.01	ns	***
C16:1 *t*6*+t*7	0.01	0.01	ns	†
C16:1 *t*8*+t*9	0.13	0.13	ns	***
C16:1 *t*11*+t*12	0.04	0.04	ns	**
C17:1 *c*9	0.18	0.18	ns	***
C17:1 *t*9	0.027	0.031	**	***
C18:1 *t*5	0.01	0.01	ns	ns
C18:1 *t*6–8	0.19	0.19	ns	ns
C18:1 *t*9	0.26	0.27	ns	ns
C18:1 *t*10	0.29	0.28	ns	ns
C18:1 *t*11	1.19	1.22	ns	**
C18:1 *t*12	0.20	0.20	ns	ns
C18:1 *t*13–14 + *c*6–8	0.35	0.35	ns	ns
C18:1 *c*9 + *c*10 + *t*15	15.60	16.39	*	***
C18:1 *c*11	0.28	0.30	*	*
C18:1 *c*12	0.15	0.14	ns	**
C18:1 *c*13	0.03	0.03	ns	ns
C18:1 *t*16	0.19	0.19	ns	ns
C18:1 *c*14	0.06	0.07	**	**
C20:1 *t*6–11	0.008	0.009	*	**
C20:1 *c*9 + *t*14	0.01	0.01	ns	**
C20:1 *t*15*+t*16	0.02	0.03	ns	*
C20:1 *c*11	0.03	0.03	ns	ns
Σ MUFA	20.38	21.41	*	**
Σ C18:1	18.86	19.69	†	***
Σ C18:1 *t*	2.67	2.70	ns	ns
DI_14_^c^	0.02	0.02	ns	***
DI_16_^c^	0.02	0.02	ns	**
DI_18_^c^	2.02	2.32	ns	ns

*CTRL, control diet; CBS, cocoa bean shell diet; DT, dietary treatment; SD, sampling date; c, cis; t, trans; MUFA, monounsaturated fatty acids: DI, desaturase index*.

a *Total number of samples for each group equal to 44 (11 goats × 4 sampling dates)*.

b Probability: ***P ≤ 0.001; **P ≤ 0.01; *P ≤ 0.05; ^†^0.05 < P ≤ 0.10; ns, not significant (P > 0.05). The effect of the interaction between dietary treatment and sampling date (DT × SD) was not significant; therefore, significance is only presented for the main effects.

c *Desaturase indexes. DI_14_: C14:1 c9/C14:0; DI_16_: C16:1 c9/C16:0; DI_18_: C18:1 c9/C18:0*.

Very few changes were observed when considering milk PUFA (Table 6). No differences were found for total PUFA, total octadecadienoic acids, total trans-octadecadienoic acids, total conjugated linoleic acid (CLA), and total n6 FA between the experimental groups. The total n3 FA tended to be higher in the milk from CBS-fed goats (*P* = 0.06), which led to a significant lower n6/n3 FA ratio in the milk (*P* ≤ 0.05). The majority of individual PUFA remained unaffected by the dietary treatment. Only C18:2 t11c15 and C18:2 t9c11 (which coeluted with C21:0) showed significantly higher concentration in the CBS when comparted to the CTRL group. Linoleic acid tended to be negatively affected by the inclusion of CBS in the goat diet (−4.7%, *P* = 0.09).

**Table 6:** Polyunsaturated fatty acids (g/kg fat) in milk of goats fed the control (CTRL) and cocoa bean shell (CBS) diets^a^.

**Fatty acid (g/kg fat)**	**Dietary treatment**		**Effects^b^**	
	**CTRL**	**CBS**	**DT**	**SD**
C18:2 *t, t*-NMID	0.021	0.024	†	ns
C18:2 *t*9*t*12	0.01	0.01	ns	ns
C18:2 *c*9*t*13 + *t*8*c*12	0.16	0.16	ns	**
C18:2 *t*8*c*13	0.11	0.12	ns	ns
C18:2 *c*9*t*12^c^	0.08	0.08	ns	**
C18:2 *t*9*c*12	0.02	0.02	ns	***
C18:2 *t*11*c*15	0.03	0.04	***	**
C18:2 *c*9*c*12	2.32	2.21	†	*
C18:2 *c*9*c*15	0.01	0.01	ns	**
C18:3 *c*6*c*9*c*12	0.03	0.03	ns	**
C18:3 *c*9*c*12*c*15	0.37	0.40	ns	*
CLA *c*9*t*11 + *t*7*c*9 + *t*8*c*10	0.64	0.64	ns	ns
CLA *t*9*c*11^d^	0.02	0.03	***	**
CLA *t*11*c*13 + *c*9*c*11	0.01	0.01	ns	**
CLA *t*10*c*12	0.00	0.00	ns	***
CLA *t*9*t*11	0.02	0.02	ns	***
C20:4 n6	0.10	0.10	ns	ns
C20:5 n3	0.02	0.02	ns	ns
C22:5 n3	0.02	0.03	ns	*
C22:6 n3	0.01	0.01	ns	**
Σ PUFA	4.00	3.94	ns	ns
Σ 18:2	3.44	3.34	ns	ns
Σ 18:2 *trans*	1.12	1.15	ns	ns
Σ CLA	0.69	0.71	ns	ns
Σ n3	0.53	0.57	†	ns
Σ n6	0.59	0.57	ns	ns
Σ n6/Σ n3	1.14	1.03	*	*

*CTRL, control diet; CBS, cocoa bean shell diet; DT, dietary treatment; SD, sampling date; c, cis; t, trans; NMID, non-methylene interrupted diene; MID, methylene interrupted diene; PUFA, polyunsaturated fatty acids; CLA, conjugated linoleic acids*.

a *Total number of samples for each group equal to 44 (11 goats × 4 sampling dates)*.

b Probability: ***P ≤ 0.001; **P ≤ 0.01; *P ≤ 0.05; ns, ^†^0.05 < P ≤ 0.10; not significant (P > 0.05). The effect of the interaction between dietary treatment and sampling date (DT × SD) was not significant; therefore, significance is only presented for the main effects.

c *Coeluted with C18:1 c16 + c,c-MID*.

d *Coeluted with C21:0*.

The sampling date significantly affected many of the detected individual FA and groups of FA.

The DT × SD interaction was not significant for the considered parameters.

## Discussion

### Chemical Composition of Cocoa Bean Shell

In accordance with the results of published literature, the major component in CBS was fiber (aNDFom represented almost 500 g/kg DM) (25, 53). As most of the lipids are removed during the industrial process for cocoa butter production, EE is only a minor component of CBS, showing much lower concentration particularly when compared to the EE content of CB (about 50% DM) (7). The CP content of the CBS used in this trial fell within the ranges reported in the literature [10.3 g/100 g to 27.4 g/100 g of the CBS dried weight (7)]. Despite the good CP amount of CBS, almost the 44% of total protein is composed of fraction C according to the Cornell Net Carbohydrate and Protein System (CNCPS) protein fractionation scheme (38). This fraction is represented by protein associated with lignin, tannin-protein complexes, and Maillard products, being characterized by null ruminal degradation rates and intestinal digestibility (54), thus limiting the potential application of CBS as alternative protein source in animal nutrition. High relative proportions of protein fraction C were also reported for other by-products of the agro-industry or biodiesel production chain with potential application in feed formulations, such as the hazelnut skin (55), custard apple and soursop seed cakes (56), and peanut cake (57).

Concerning the FA composition of CBS, few data are currently available in the literature. A detailed FA profile was recently reported by Botella-Martínez et al. (10) for Ghanaian CBS flours of different particle size. The obtained results confirm a prevalence of SFA in CBS, especially stearic and palmitic acids with approximately equal concentrations, followed by MUFA, with a clear prevalence of oleic acid. Similar findings were also reported by Lessa et al. (58) and Soares et al. (59) for Brazilian roasted CBS and CBS fat extract, respectively.

Cocoa bean shell is a good source of phenolic compounds, which are responsible for its bio-functional properties. Several authors investigated the polyphenolic fraction of CBS, and high variability has been shown (i.e., 6.04–94.95 mg of gallic acid equivalents and 1.70–25.30 mg of catechin equivalents/g of dried CBS for total phenols and total tannins, respectively) according to the polyphenolic extraction conditions (7, 53). Because of the use of different extraction methods and/or different units of measure used for the expressions of the obtained results, it is difficult to compare the obtained results with those obtained by other authors. However, with comparable analytical methods used in the current trial, Campione et al. (38) found amounts of TEP in CBS equal to 43.9 g/kg DM, about the 70% of which were tannins. Such values are about 4.8 times higher than those found for the CBS used in the current trial. However, it has been demonstrated that various factors concur to affect the polyphenolic concentration of CBS, such as plant genotype, geographical origin, harvest season, stress conditions for the plant and, finally, cocoa manufacturing processes, the latter being able to cause polyphenol degradation (7). The presence of polyphenolic compounds is typical of many agro-industrial by-products. The TEP concentration of CBS is lower if compared to that of other by-products shown to be potentially used as raw materials in feed formulations, such as hazelnut skin (265 g/kg DM) (51) or pistachio hulls (103.0 g/kg DM) (60).

The theobromine content of the CBS used in this trial (4.3 g/kg DM) is in line with lower-range values reported in the literature for this by-product (31) and slightly lower that the theobromine content of the CBS (7.2 g/kg DM) or cocoa pod husk (6.9 g/kg DM) tested for their suitability in sheep nutrition by Campione et al. (38) and Carta et al. (61), respectively. The above-mentioned theobromine levels are also lower than those reported for hot water treated CBS by other Authors (30).

### Dry Matter Intake, Milk Yield, and Milk Main Constituents

The inclusion of CBS in the diet of the goats negatively impaired their total DMI (Table 2). Lots of agro-industrial by-products are characterized by low overall nutritional quality; in addition, some of them contain antinutritional factors that negatively affect palatability (3). However, in the current study, the reduction of the DMI only regarded the roughage part of the diet, as the concentrate was always fully eaten by the animals of both experimental groups (Table 2). This demonstrates that a CBS inclusion level of 17% in the concentrate feed does not cause palatability issues in the caprine species. In cattle and sheep, dietary tannins can create complexes with lignocellulose, inhibiting cellulolytic microorganisms and fibrolytic enzymatic activity, and increasing rumen physical filling, with negative consequences on voluntary DMI and fiber digestion (62, 63). However, goats and other browsing animal species are able to produce tannin-binding saliva and stimulate the proliferation of tannin-tolerant bacteria, mostly overcoming the negative effects of tannins on intake and nutrient digestion (64, 65). da Silva et al. (66) recently demonstrated that a dietary concentration of CT equal to 5% DM did not affect the DMI and nutrient intake of goats from different genotypes. Concerning voluntary DMI, no adverse effects are expected in goats fed diets containing up to 10% DM of CT (65). Having this in mind and given the relatively low concentration of tannins in the CBS used in the current trial trial (both TT and CT lower than 0.1% DM for the CBS diet), it is unlikely that the reduction of the voluntary DMI in the CBS-fed goats may be imputed to the dietary tannin concentration. As a confirmation for such a hypothesis, ewes fed alfalfa hay *ad libitum* and 800 g of conventional or 11.7% CBS-containing concentrate (dietary TT equal to 0.4% DM) showed no significant alterations of their voluntary DMI (38). However, when feeding growing goats (18–20 months of age) with a diet containing up to 50% CBS or cocoa dust, Aregheore (67) observed in both cases a reduction of the voluntary DMI by the animals, which consequently led to a lower body weight gain, when compared to growing goats of comparable age and initial body weight gain fed dried brewers' grain as the control group. The author attributed the lower production performance of the goats fed CBS and cocoa dust to the presence of theobromine in the cocoa by-products. Unfortunately, Aregheore (67) did not report the theobromine level of the CBS. However, considering an average theobromine concentration in the CBS equal to 13 g/kg, it can be estimated that the theobromine intake in Aregheore (67) was 6.9 g/growing goat per day, corresponding to 290 mg/kg body weight per day (31). As the dietary inclusion level of CBS was much lower in the present study, the theobromine intake was also much lower, corresponding to an average value of about 1.51 g/head × day. From these considerations, an issue arises about the identification of the maximum level of ingested theobromine capable of negatively influence the voluntary DMI in adult goats. Further studies are necessary to clarify this issue.

In this study, milk yield as well as the contents and yields of milk fat, protein and casein were not affected by the dietary inclusion of CBS, confirming the results obtained in CBS-fed and cocoa husks-fed ewes by Campione et al. (38) and Carta et al. (61), respectively. Consistently, Correddu et al. (68) showed that, in general, the dietary inclusion of agro-industrial by products only showed negligible effects on milk yield and composition in small ruminants. The milk urea contents obtained in the current study fell within range values reported in literature for dairy goats (69). However, differently from other milk macro components, but similarly to what observed by Campione et al. (38) in dairy sheep, milk from the CBS-fed goats showed a significantly lower amount of urea when compared to the CTRL group. Campione et al. (38) hypothesized that such a result could be the consequence of the intrinsic characteristics of proteins in CBS. In fact, the high relative proportion of by-pass protein (rumen undegradable protein equal to 59% of total CP in the CBS used in the present study study), and particularly of CNCPS fraction C (unavailable nitrogen) (38), lowers the ratio between available protein and available energy at ruminal level in the CBS-fed animals, even though the CTRL and CBS diets were formulated to be isonitrogenous and isoenergetic. Such results confirm previous findings by Giaccone et al. (70), who showed seasonal variations in the urea content of milk from Girgentana goats, with higher values associated with grazing activities in winter months, when pastures are richer in soluble nitrogen and poorer in NDF and energy.

Furthermore, the observed significant reduction of milk lactose in the CBS-fed group of goats is consistent with the findings of Cobanović et al. (71) who showed a clear positive correlation between urea and lactose contents in goat milk.

Finally, the slight differences observed in yield and quality of milk, and dry matter intake (although the latter statistically significant) did not affect feed efficiency (Table 3). The obtained results are consistent with previous findings in literature: feed efficiency did not vary when different by-products are fed to dairy goats (72, 73). Moreover, the FE results are comparable with data obtained in a previous experimental trial in the same farm (74).

### Milk Fatty Acid Profile

Only few changes were detected in the milk FA profile between the two experimental groups. Dietary tannins have been shown to modulate specific metabolic pathways and steps of the rumen biohydrogenation of dietary lipids (75), leading to an improved quality of the lipid fraction of milk in small ruminants (76). However, in the current study, the lack of significant changes between the two experimental groups in the milk concentrations of trans-octadecenoic and trans-octadecadienoic acids, which are intermediate products of ruminal biohydrogenation of dietary unsaturated fatty acids, suggests a lack or only negligible effects of tannins in CBS on the rumen microflora composition and activity. This may be associated to the relatively low amounts of tannins in CBS, when compared to the amounts of tannins reported for other agro-industrial by-products (68). Most probably, the amount of TT in the CBS-containing diet in the current trial was not great enough to significantly affect rates and extents of ruminal biohydrogenation in dairy goats, as already previously suggested by other authors (46, 77). Lignin and other non- or slowly degradable fiber fractions of feeds can significantly slow down the passage rates of both solid and liquid matter in the rumen (78). This led Campione et al. (38) hypothesize that the high concentration of ADL in CBS could have increased the retention time of the feed inside the reticulo-rumen, consequently increasing the time available for the microflora to biohydrogenate the unsaturated fatty acids ingested with the diet, therefore hiding the effects of tannins on rumen biohydrogenation. Such hypothesis may be also coherent to the observed reduced DMI of CBS-fed goats in the current trial, as increasing retention times also lead to increased rumen physical filling (78).

Milk odd-chain FA and BCFA mainly derive from rumen bacteria. Despite their low concentrations in ruminant milk fat, these FA of microbial origin have aroused interest among nutritionists as diagnostic parameters of rumen function (79) and because of their putative beneficial health effects in humans (80). In the current trial, milk from the CBS-fed goats showed increased concentrations for the majority of both *iso* and *anteiso* BCFA forms. Ruminant diet is addressed as the main factor affecting the concentration of BCFA in milk fat (81). As dietary tannins negatively affect the concentration of BCFA in milk (51), the increased BCFA concentration in the CBS-fed goats strengthen the hypothesis that the tannin level of the CBS diet was too low to exert detrimental effects on the rumen metabolism in this species. When compared to the CTRL-fed goats, the CBS-fed goats ingested lower amounts of PUFA from the diet (Table 2). As PUFA are known to exert toxic effects on the rumen microflora, particularly on cellulolytic bacteria, a reduced PUFA intake from the diet may have determined a quantitative increase of bacteria biomass inside the rumen (82), and a subsequent higher availability of BCFA for milk fat synthesis in the mammary gland (83). In addition, the higher fiber content of the CBS diet (Table 1) may also partly explain the observed increase of BCFA in the CBS-fed goats, as a positive association has been previously reported between dietary fiber and FA of microbial origin (83). Consistently, Nudda et al. (83) recently reported significant seasonal variations in the BCFA concentrations in goat milk and cheese fat mainly as a consequence of reduced contents of PUFA (mainly ALA) and protein, and increased fiber, in grazed pastures of advanced phenological stages. To the best of our knowledge, to date no studies are available on the effects of the dietary inclusion of cocoa by-products on the compositional variation of rumen microbiome to better understand the biological mechanisms involved in the observed significant BCFA increase in milk fat from the CBS-fed goats.

Being CBS rich in stearic and oleic acids (Table 1), the intake of both these FA was significantly higher in the CBS-fed goats (Table 2), which led to an increase in the concentration of both stearic and oleic acids in milk fat (Tables 4, 5). The presence of stearic acid in milk is a consequence of both preformed forms ingested with the diet and forms deriving from the complete ruminal biohydrogenation of dietary unsaturated fatty acids, including oleic acid (84). Therefore, in this trial it is presumable to assume that both the above-mentioned origin sources were reasons for the increased concentration of stearic acid in milk from the CBS-fed goats. In turn, oleic acid can also derive from desaturation of stearic acid within the mammary gland thanks to the activity of the SCD enzyme (85). The SCD activity is inhibited, through transcriptional or post-transcriptional mechanisms, by the availability of rumen by-pass PUFA and trans intermediates of ruminal biohydrogenation (86), the concentrations of which were not affected by the inclusion of CBS in the goat diet. This led to a similar activity of SCD in both groups of goats, as estimated by the calculation of different desaturase indexes (74, 81, 87). When feeding dairy ewes with diets containing CBS, Campione et al. (38) detected significantly higher rumen contents of both stearic and oleic acids, which led to higher contents of these FA in ovine milk and cheese. The observed increase of oleic acid in milk can be considered a positive effect of the inclusion of CBS in the goats' diet. In fact, oleic acid presents beneficial modulatory effects on various diseases including cancer, autoimmune- and inflammatory-related pathologies (88).

Even if there is no consensus nowadays in the scientific community on what the ∑ n6/∑ n3 FA ratio of the diet should be to obtain optimal health effects in humans (89), it is well-known that it can affect inflammation and other biological processes (90, 91). A recommended value equal or lower than four was suggested in the past for the human diet (92). In the current study, the milk from the CTRL group fell within this range, with a value (∑ n6/∑ n3 FA ratio equal to 1.14) comparable to that of ancestral human diets (93). A further significant improvement (∑ n6/∑ n3 FA ratio equal to 1.03) was obtained when CBS was included in the goat diet, supporting the findings of Campione et al. (38) with the administration of CBS in the diet of dairy ewes.

In conclusion, we showed that a possible utilization to upgrade the value of by-products from the cocoa industry, such as the CBS, is their inclusion in diets for livestock animals. Particular attention by nutritionists must be addressed to the presence of theobromine in CBS. The observed reduction of the DMI by the goats may be associated to the presence of this alkaloid or high amount of lignin in CBS and needs further considerations. However, if CBS is included in diets for lactating goats in amounts that fall within the limits imposed by the current legislation for theobromine, no adverse effects are expected on the milk production performance, particularly when considering milk yield and the contents of milk main constituents. An expected outcome of the high proportion of unavailable nitrogen in CBS is the reduction of the urea content in goat milk. When considering goat milk fatty acids, only minor changes were induced by CBS, including increased concentrations of BCFA, stearic and oleic acids, and a reduced ∑ n6/∑ n3 FA ratio. Despite the presence of tannins in CBS, no impairments of ruminal biohydrogenation pathways or steps were observed.

## Data Availability Statement

The raw data supporting the conclusions of this article will be made available by the authors, without undue reservation.

## Ethics Statement

According to the National Italian Law (D.L. 26/2014), no specific ethical approval is required for this study, as no pain, suffering, distress or prolonged damage equivalent to or greater than that caused by the insertion of a needle was applied. Written informed consent was obtained from the breeder for the participation of his animals in this study.

## Author Contributions

PC, AM, CL, and MR conceived and designed the experiment, analyzed, and interpreted the data. PC, AM, and LC prepared the diets, performed the trial, and collected the experimental data. CL, VM, and LC performed the chemical analyses. PC performed the statistical analysis. MR, PC, AM, and LC analyzed and interpreted the data. MR and LC wrote the first draft of the manuscript. All authors critically reviewed the manuscript for its intellectual content and gave their approval for the final version to be published.

## Funding

This research was funded by the University of Turin (Project CORP_RILO_17_01—*Valorizzazione di by-products nell'alimentazione animale: da scarto a functional feeds*).

## Conflict of Interest

The authors declare that the research was conducted in the absence of any commercial or financial relationships that could be construed as a potential conflict of interest.

## Publisher's Note

All claims expressed in this article are solely those of the authors and do not necessarily represent those of their affiliated organizations, or those of the publisher, the editors and the reviewers. Any product that may be evaluated in this article, or claim that may be made by its manufacturer, is not guaranteed or endorsed by the publisher.

## Abbreviations

ADFom: acid detergent fiber
ADL: acid detergent lignin
aNDFom: neutral detergent fiber
BCFA: branched chain fatty acids
*c*: *cis*
CB: cocoa beans
CBS: cocoa bean shell
CLA: conjugated linoleic acid
CNCPS: Cornell Net Carbohydrate and Protein System
CP: crude protein
CT: condensed tannins
CTRL: control
DI: desaturase index
DM: dry matter
DMI: dry matter intake
DT: dietary treatment
ECM: energy corrected milk
EE: ether extract
FA: fatty acid
FAME: fatty acid methyl esters
FE: feed efficiency
HT: hydrolysable tannins
MID: methylene interrupted diene
MUFA: monounsaturated fatty acids
n: number of samples
n.d.: not detected
NE_L_: net energy for lactation
NMID: non-methylene interrupted diene
ns: not significant
NSC: non-structural carbohydrates
NTP: non-tannin phenols
PUFA: polyunsaturated fatty acids
RDP: rumen degradable protein
SCC: somatic cell count
SD: sampling date
SFA: saturated fatty acids
*t*: *trans*
TEP: total extractable phenols
TFA: total fatty acids
TT: total tannins.
